# Lateral Lymph Node Metastases in Locally Advanced Low Rectal Cancers May Not Be Treated Effectively With Neoadjuvant (Chemo)Radiotherapy Only

**DOI:** 10.3389/fonc.2019.01355

**Published:** 2019-12-03

**Authors:** Anouck Haanappel, Hidde M. Kroon, Dennis P. Schaap, Sergei Bedrikovetski, Nagendra N. Dudi-Venkata, Hong X. Lee, Michelle L. Thomas, Jianliang Liu, Maxime J. M. van der Valk, Harm J. T. Rutten, Geerard L. Beets, Miranda Kusters, Tarik Sammour

**Affiliations:** ^1^Colorectal Unit, Department of Surgery, Royal Adelaide Hospital, Adelaide, SA, Australia; ^2^Department of Surgery, Amsterdam University Medical Centers, Amsterdam, Netherlands; ^3^Department of Surgery, Catharina Hospital, Eindhoven, Netherlands; ^4^Discipline of Surgery, Faculty of Health and Medical Science, School of Medicine, University of Adelaide, Adelaide, SA, Australia; ^5^Department of Surgery, Leiden University Medical Center, Leiden, Netherlands; ^6^GROW, School of Oncology and Developmental Biology, University of Maastricht, Maastricht, Netherlands; ^7^Department of Surgery, The Netherlands Cancer Institute-Antoni van Leeuwenhoek, Amsterdam, Netherlands

**Keywords:** lateral lymph nodes, locally advanced low rectal cancer, neoadjuvant (chemo)radiotherapy, oncological outcomes, survival

## Abstract

**Background:** In the West, pre-treatment abnormal lateral lymph nodes (LLN+) in patients with a low locally advanced rectal cancer (AJCC Stage III), are treated with neoadjuvant (chemo)radiotherapy (nCRT), without a lateral lymph node dissection (LLND). It has been suggested, however, that LLN+ patients have higher local recurrence (LR) rates than similarly staged patients with abnormal mesorectal lymph nodes only (LLN−), but no comparative data exist. Therefore, we conducted this international multi-center study in the Netherlands and Australia of Stage III rectal cancer patients with either LLN+ or LLN− to compare oncological outcomes from both groups.

**Materials and Methods:** Patients with Stage III low rectal cancer with (LLN+ group) or without (LLN− group) abnormal lateral lymph nodes on pre-treatment MRI were included. Patients underwent nCRT followed by rectal resection surgery with curative intent between 2009 and 2016 with a minimum follow-up of 2-years. No patient had a LLND. Propensity score matching corrected differences in baseline characteristics.

**Results:** Two hundred twenty-three patients could be included: 125 in the LLN+ group and 98 in the LLN− group. Between groups, there were significant differences in cT-stage and in the rate of adjuvant chemotherapy administered. Propensity score matching resulted in 54 patients in each group, with equal baseline characteristics. The 5-year LR rate in the LLN+ group was 11 vs. 2% in the LLN− group (*P* = 0.06) and disease-free survival (DFS) was 64 vs. 76%, respectively (*P* = 0.09). Five-year overall survival was similar between groups (73 vs. 80%, respectively; *P* = 0.90).

**Conclusions:** In Western patients with Stage III low rectal cancer, there is a trend toward worse LR rate and DFS rates in LLN+ patients compared to similarly staged LLN− patients. These results suggest that LLN+ patients may currently not be treated optimally with nCRT alone, and the addition of LLND requires further consideration.

## Introduction

Local recurrences (LR) in patients who have previously been treated curatively for locally advanced low rectal cancer [American Joint Committee on Cancer (AJCC) Stage III] are associated with severe morbidities such as pain and reduced quality of life. Over the past three decades, the introduction of neoadjuvant (chemo)radiotherapy (nCRT) and the broad application of rectal resections according to the principle of total mesenteric excision (TME) have reduced 5-year LR rates to 5–10% (1–3). However, it has been suggested that patients with pre-treatment abnormal lateral lymph nodes (LLN+), which are present in approximately 15–20% of patients with Stage III rectal cancer, still have increased LR rates (4–6).

With the aim of reducing LR rates, treatment strategies for patients with LLN+ have evolved differently around the world. In the West, treatment consists of nCRT followed by TME surgery, typically without resecting the LLN+ (7). In contrast, in the East, the standard treatment is surgery combining TME with a lateral lymph node dissection (LLND), often without nCRT (8).

Interestingly, despite these differences in treatment approach, comparable LR rates have been reported: 6.9% in Eastern patients undergoing TME and LLND, and 5.8% in Western patient receiving nCRT and TME (9) while others have suggested favorable results following the Eastern approach (10, 11). Furthermore, some studies demonstrated that LLN+ cannot be eradicated completely by nCRT only, suggesting that the Western treatment may not be sufficient for local disease-control (12–14). On the other hand, a LLND is a complex surgical procedure with some risks of post-operative complications and long-term morbidity such as sexual and urinary dysfunction (15, 16). Therefore, if not oncologically necessary, it may be in the patient's best interest to omit this procedure. In addition, it is unclear whether combining nCRT and LLND adds anything over each alone.

Current knowledge about the behavior of LLN+ as distinct from mesorectal node positivity (N+) is inconclusive. There is only one small single-center study available looking at oncological outcomes following treatment of the two subcategories of Stage AJCC III patients: patients with LLN+ compared to those with abnormal mesorectal lymph nodes only (LLN−) (17). Therefore, this international multi-center cohort study was conducted to compare long-term oncological outcomes of patients with LLN+ and LLN−, who were both treated the same according to the Western protocol of nCRT followed by TME surgery.

## Patients and Methods

The current study was conducted at three hospitals in the Netherlands (Antoni van Leeuwenhoek-Netherlands Cancer Institute in Amsterdam, Catharina Hospital in Eindhoven and Leiden University Medical Center in Leiden) and two hospitals in Australia (Royal Adelaide Hospital and St Andrew's Hospital, both in Adelaide).

Included were patients of 18 years of age and over, who were treated with curative intent between 2009 and 2016 for a low (within 8 cm of the anal verge) AJCC clinical Stage III locally advanced rectal cancer with abnormal mesorectal lymph nodes with or without LLN+ on pre-treatment magnetic resonance imaging (MRI). All patients had pre-treatment abnormal mesorectal lymph nodes on MRI imaging and/or LLN+ in the obturator, internal iliac, external iliac, and/or common iliac basins. Abnormal mesorectal lymph nodes and LLN+ were defined as nodes with a short-axis of ≥5 mm on MRI with or without malignant features (10, 14, 18). In order to identify eligible patients, during the study period pelvic MRI scans for rectal cancer were re-reviewed by senior radiologists at each site. All patients underwent nCRT followed by TME surgery. Excluded were patients with concurrent distant metastatic disease in para-aortic lymph nodes or distant organs at the time of diagnosis, those with previous radiotherapy (RT) to the pelvis precluding nCRT, and those who did not undergo TME surgery. The study was approved by the human research ethics committee at each site.

After tissue diagnosis, all patients were clinically staged according to the 7th edition of the AJCC Colon and Rectum Cancer Staging and discussed the local multidisciplinary team meetings (MDT) (4). Neoadjuvant therapy was conducted according to the hospital's local protocol and consisted of either short-course RT (5 × 5 Gray) or long-course CRT (45–50.4 Gray) applied in 28 fractions over 7 weeks applied to the pelvis with individually shaped portals and the use of a three-field or four-field box technique with concomitant one of the following chemotherapy regimens: FOLFOX (folinic acid, fluorouracil, and oxaliplatin) on the first day of each week of RT, daily oral capecitabine, or 5-fluorouracil for five 2-week cycles. For LLN+ patients, it was confirmed for each center that standard practice involved including the obturator and internal iliac compartments in the irradiated field. Following completion of nCRT, all patients underwent TME surgery with curative intent by means of a low anterior resection (LAR), an abdominoperineal resection (APR) or a pelvic exenteration in case of involvement of adjacent organs. Post-operative histopathological staging was performed on the surgical specimen. The patient was then again discussed at the MDT where consensus was reached for adjuvant treatment and follow-up. Adjuvant chemotherapy consisted of FOLFOX, 5-fluorouracil, capecitabine or capecitabine+oxaliplatin (CAPOX) for three 6–8 week cycles. LR were defined as tumor regrowth in the lower pelvis at the anastomotic site, at the site of the previously resected mesorectal tissues, or in the lateral compartment.

Included patients were divided into two groups: a LLN+ and a LLN− group (all stage III). Continuous variables are shown as medians with range, and categorical variables are presented as absolute numbers with percentages. Differences in characteristics between the LLN+ and the LLN− group were evaluated with the Mann Whitney *U*-test for continuous variables, and the Chi-square or the Fisher's exact test for categorical variables (19). LR-free survival (LRFS: defined as recurrent disease in the pelvis), distant metastatic-free survival (DMFS: defined as recurrent disease distally), disease-free survival (DFS: defined as recurrent disease anywhere), and overall survival (OS; defined as death due to any cause) were estimated using the Kaplan-Meier method, with the log-rank test from the day of surgery until detection of LR, distant metastases or date of death (20). To minimize the effect of confounding factors on the outcome between both groups, a propensity score matching was performed for the covariates gender, clinical tumor (cT)-stage, resection margin, adjuvant chemotherapy, and follow-up time. LLN+ patients in whom selected lateral lymph nodes were harvested were excluded from the propensity score matching. A *p* ≤ 0.05 was considered statistically significant. Statistical analyses were performed using SPSS version 25.0 (IBM Corp, Armonk, NY, USA) and GraphPad Prism version 8.0.2 (GraphPad Software Inc., San Diego, CA, USA).

## Results

### Complete Cohort

Baseline patient and tumor characteristics are shown in Table 1. A total of 223 patients with Stage III rectal cancer were included: 125 patients in the LLN+ group (lateral lymph node size range 5.1–48.0 mm), and 98 in the LLN− group. There was a significant difference in median height of the tumor from the anal verge on MRI: 3.2 cm in the LLN+ group (range 0.0–8.0 cm) and 6.0 cm in the LLN− group (range 0.1–8.0 cm, *P* < 0.0001). LLN+ patients had more cT4 disease compared to LLN− group (37 vs. 11%, *P* < 0.0001) and received more long-course CRT (83 vs. 69%, *P* = 0.02). All other baseline characteristics were similar between groups.

**Table 1:** Baseline patient and tumor characteristics.

**Characteristics**	**Complete cohort**		***P*-value**	**Matched cohort**		***P*-value**
	**LLN+ (*n* = 125)**	**LLN− (*n* = 98)**		**LLN+ (*n* = 54)**	**LLN− (*n* = 54)**	
**Age** in years, median (range)	64 (25–85)	63 (20–89)	0.76	61.5 (25–82)	64.5 (20–80)	0.24
**Gender**, *n* (%)						
Male	88 (70%)	58 (59%)	0.08	20 (37%)	20 (37%)	>0.99
Female	37 (30%)	40 (41%)		34 (63%)	34 (63%)	
**BMI**, median (range)	26.1 (16.4–46.2)^a^	25.8 (14.2–37.8)^b^	0.97	27.1 (18.8–40.8)^c^	27.3 (14.2–37.8)^d^	0.84
**MRI-height of tumor** in cm, median (range)						
	3.2 (0.0–8)	6.0 (0.1–8.0)	** <0.0001**	3.3 (0.0–7.9)	6.0 (0.1–8.0)	** <0.0001**
**cT stage on MRI**, *n* (%)						
cT2	1 (1%)	2 (2%)	** <0.0001**	1 (2%)	1 (2%)	>0.99
cT3	78 (62%)	85 (87%)		43 (80%)	43 (80%)	
cT4	46 (37%)	11 (11%)		10 (18%)	10 (18%)	
**cN stage on MRI**, *n* (%)						
cN1	61 (49%)	50 (51%)	0.74	32 (59%)	31 (57%)	0.85
cN2	64 (51%)	48 (49%)		22 (41 %)	23 (43%)	
**CRM-involvement**, *n* (%)						
No	72 (58%)	66 (67%)	0.14	34 (63%)	39 (72%)	0.30
Yes	53 (42%)	32 (33%)		20 (37%)	15 (28%)	
**Short-axis LLN+** in mm,						
median (range)	6.6 (5.0–28.0)	Not applicable	–	6.0 (5.0–26.4)	Not applicable	–
**Neoadjuvant therapy type**, *n* (%)						
Short-course RT	21 (17%)	30 (31%)	**0.015**	9 (17%)	18 (33%)	0.08
Chemoradiotherapy	104 (83%)	68 (69%)		45 (83%)	36 (67%)	

*LLN+, abnormal lateral lymph nodes; LLN−, abnormal mesorectal nodes only; BMI, body mass index; CRM, circumferential resection margin; c, clinical; RT, radiotherapy*.

a *4 patients missing*.

b *38 patients missing*.

c *1 patient missing*.

d *20 patients missing*.

*Bold P-values indicate statistically significant values*.

Low anterior resection was more frequently performed in the LLN− group (67 vs. 46% for LLN+ patients, *P* = 0.03), while LLN+ patients underwent more APRs (53 vs. 31% for the LLN− group, *P* = 0.003, Table 2). More lymph nodes were harvested in the LLN+ group (median 16 vs. 13, *P* = 0.002). In the LLN+ group, selected lateral lymph nodes were harvested in 10 patients (so-called “cherry-picking surgery”: no complete lateral lymph node dissection was performed, median nodes harvested: 1 node, range 1–3 nodes) of which in 6 tumor was harvested upon histopathological examination. Pathological TNM staging and negative resection margins (R0) were similar between groups (90 vs. 94% in the LLN+ and LLN− group, respectively, *P* = 0.28). More LLN− patients received adjuvant chemotherapy (70 vs. 29% for LLN+ patients, *P* < 0.0001).

**Table 2:** Peri-operative characteristics and post-operative histopathology.

**Characteristics**	**Complete cohort**		***P*-value**	**Matched cohort**		***P*-value**
	**LLN+ (*n* = 125)**	**LLN− (*n* = 98)**		**LLN+ (*n* = 54)**	**LLN− (*n* = 54)**	
**Type of surgery**, *n* (%)						
LAR	57 (46%)	66 (67%)	**0.003**	24 (44%)	35 (65%)	**0.001**
APR	67 (53%)	30 (31%)		30 (56%)	17 (31%)	
Exenteration	1 (1%)	2 (2%)		0 (0%)	2 (4%)	
**ypT stage**, *n* (%)						
ypT0	14 (11%)	15 (15%)	0.69	9 (17%)	13 (24%)	0.58
ypT1	7 (6%)	6 (6%)		3 (6%)	6 (11%)	
ypT2	34 (27%)	19 (20%)		13 (24%)	12 (22%)	
ypT3	56 (45%)	47 (48%)		24 (44%)	16 (30%)	
ypT4	14 (11%)	11 (11%)		5 (9%)	7 (13%)	
**YpN stage**, *n* (%)						
ypN0	75 (60%)	56 (57%)	0.48	32 (59%)	37 (68%)	0.31
ypN1	34 (27%)	33 (34%)		16 (30%)	16 (30%)	
ypN2	16 (13%)	9 (9%)		6 (11%)	1 (2%)	
**Resection margin***, n* (%)						
R0	113 (90%)	93 (94%)	0.28	54 (100%)	54 (100%)	>0.99
R1	11 (9%)	3 (4%)		0	0	
R2	1 (1%)	2 (2%)		0	0	
**Total number of lymph nodes harvested**, median (range)	16 (5–46)	13 (1–33)	**0.002**	18 (5–46)	13 (2–33)	**0.004**
**Adjuvant chemotherapy**, *n* (%)						
No	89 (71%)	28 (29%)	** <0.0001**	26 (48%)	26 (48%)	>0.99
Yes	36 (29%)	69 (70%)		28 (52%)	28 (52%)	
Unknown	–	1 (1%)		–	–	

*LLN+, abnormal lateral lymph nodes; LLN−, abnormal mesorectal nodes only; LAR, low anterior resection; APR, abdominoperineal resection; yp, pathological stage after neoadjuvant (chemo)radiotherapy. Bold P-values indicate statistically significant values*.

**Figure 1:**
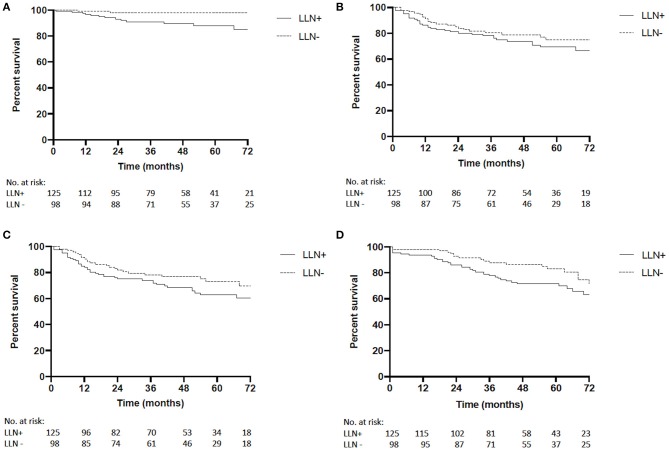
**(A)** Complete cohort local recurrence-free survival (LRFS) of LLN+ and LLN− patients [5-year LRFS 88 vs. 98%, respectively, *P* = 0.009, hazard ratio (HR) 5.72, 95%CI 2.07–15.77]. **(B)** Complete cohort distant metastatic-free survival (DMFS) of LLN+ and LLN− patients (5-year DMFS 69 vs. 75%, respectively, *P* = 0.30, HR 1.33, 95%CI 0.78–2.28). **(C)** Complete cohort disease-free survival (DFS) of LLN+ and LLN− patients (5-year DFS 63 vs. 73%, respectively, *P* = 0.15, HR 1.45, 95%CI 0.89–2.37). **(D)** Complete cohort overall survival (OS) of LLN+ and LLN− patients (5-year OS 72 vs. 83%, respectively, *P* = 0.06, HR 1.64, 95%CI 0.98–2.73).

Median follow-up time for the LLN+ group was 57 months (range 1–98) and 59 months in the LLN− group (range 1–99; *p* = 0.55). The 5-year LRFS was significantly shorter in the LLN+ group compared to the LLN− group [88 vs. 98%, *P* = 0.009, hazard ratio (HR) 5.72, 95%CI 2.07–15.77, Figure 1A]. No differences between groups were seen in the 5-year DMFS (69 vs. 75%, *P* = 0.30, HR 1.33, 95%CI 0.78–2.28, Figure 1B), or DFS (63 vs. 73%, *P* = 0.15, HR 1.45, 95%CI 0.89–2.37, Figure 1C). Five-year OS in LLN+ patients was 72% and was 83% in the LLN− group (*P* = 0.06, HR 1.64, 95%CI 0.98–2.73, Figure 1D).

### Matched Cohort

After propensity score matching, 54 patients remained in each group. In the matched cohort, age, gender, body mass index (BMI), cT-stage, clinical nodal (cN) stage, pre-treatment tumor involvement of the circumferential resection margin and nCRT were similar between both groups (Table 1). The distance of the tumor from the anal verge remained significantly different with a median of 3.3 cm (range 0.0–7.9 cm) in the LLN+ group compared to 6.0 cm in LLN− patients (range 0.1–8.0 cm, *p* < 0.001).

The majority of patients in the LLN+ group underwent an APR while LAR remained the most performed procedure in the LLN− group (56 vs. 65%, *P* = 0.001). More lymph nodes were harvested in LLN+ patients (median 18 vs. 13 for LLN− patients, *P* = 0.004). The number of patients receiving adjuvant chemotherapy was similar between both groups after matching for pre-operative variables (*p* > 0.99). All other peri-operative characteristics and post-operative histopathological findings were similar between both groups (Table 2).

**Figure 2:**
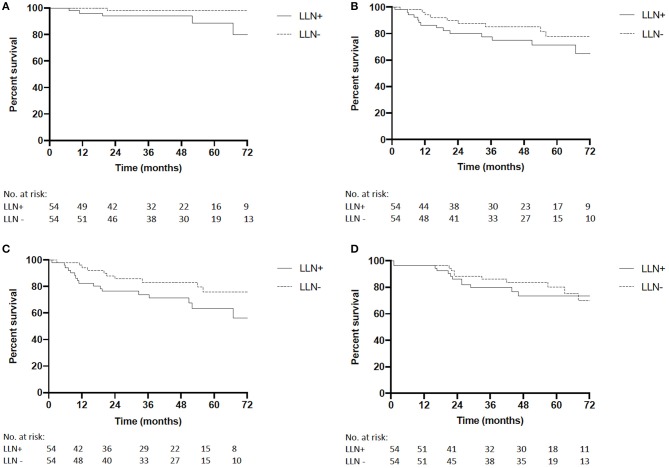
**(A)** Matched cohort local recurrence-free survival (LRFS) of LLN+ and LLN− patients (5-year LRFS 89 vs. 98.0%, respectively, *P* = 0.07, HR 5.89, 95%CI 0.91–22.87). **(B)** Matched cohort distant metastatic-free survival (DMFS) of LLN+ and LLN− patients (5-year DM 71.2 vs. 77.8%, respectively, *P* = 0.21, HR 1.70, 95%CI 0.75–3.85). **(C)** Matched cohort disease-free survival (DFS) of LLN+ and LLN− patients (5-year DFS 63.5 vs. 76.0%, respectively, *P* = 0.09, HR 1.96, 95%CI 0.92–4.17). **(D)** Matched cohort overall survival (OS) of LLN+ and LLN− patients (5-year OS 73.5 vs. 80.1%, respectively, *P* = 0.90, HR 1.05, 95%CI 0.48–2.27).

In the matched cohort, median follow-up time was 58 months in the LLN+ group (range 1–98) and 60 months in the LLN− group (range 1–99; *p* = 0.61). Five-year LRFS was 89% in LLN+ patients and 98% in LLN− patients (*P* = 0.07, HR 5.89, 95%CI 0.91–22.87, Figure 2A). During follow-up, four of the LLN+ patients had a lateral LR and one had a central LR, while of the LLN− patients, one central and no lateral LR were observed. One LLN+ patient developed a synchronous lateral LR and distant metastases to the liver at 67 months. One LLN+ patient had metachronous recurrent disease with a lateral LR at 19 months and distant metastases to the liver at 21 months. The other patients with LR in either group (*n* = 4) did not develop distant metastases. Five-year DMFS was 71% in LLN+ patients vs. 78% in LLN− patients (*P* = 0.21, HR 1.70, 95%CI 0.75–3.85, Figure 2B) and 5-year DFS was 64% in LLN+ patients and 76% in LLN− patients (*P* = 0.09, HR 1.96, 95%CI 0.92–4.17, Figure 2C). Finally, 5-year OS was similar between both groups (74% for LLN+ vs. 80% for LLN− patients, *P* = 0.90, HR 1.05, 95%CI 0.48–2.27, Figure 2D).

## Discussion

The current study suggests that patients who suffer from an AJCC stage III low rectal cancer with LLN+ have a worse LRFS and DFS, both showing a trend toward significance compared to similarly staged LLN− patients. These results suggest that in the West rectal cancer patients with LLN+ may not be treated optimally.

Metastases to the lateral lymph nodes in patients with Stage III low rectal cancer occur via extra-mesorectal spread (5, 21). This means that these LLN+ are not resected during standard TME surgery and are therefore a potential site for LR (6, 22). When these patients are treated with surgery only, LLN+ patients have worse survival rates compared to those with mesorectal lymph node metastases only (22–25). For this reason, LLN+ patients in the East, mainly Japan, undergo a LLND at the time of TME surgery, however, without undergoing nCRT in most cases (8). In contrast, Western patients with LLN+ are treated similarly to LLN− patients, both with nCRT, increasing the RT field to include the lateral nodal basins in most LLN+ patients, followed TME surgery without performing a LLND. It has previously been suggested that this Western approach may result in worse oncological outcomes, mainly LR rates, for Stage III patients with LLN+ compared to similarly staged LLN− patients, but comparative data have been scarce thus far (1–3, 10, 17, 26).

After analyzing the complete cohort, there were some significant baseline differences between both groups, likely causing a bias in the long-term oncological results. Therefore, we performed a propensity score matching for gender, cT-stage and adjuvant chemotherapy with two similar groups in baseline characteristics (27).

In the matched cohort, there was a clinically relevant (though non-significant) trend toward worse 5-year LRFS and DFS in LLN+ patients compared to LLN− patients. The absolute differences were 9 and 12%, respectively (*P* = 0.07 and *P* = 0.09). Similar findings have been reported by Ogura et al. (26). Although they analyzed a larger cohort of patients in their study, they included patients both from the East and the West treated by a variety of different strategies, making it difficult to draw firm conclusions on the oncological behavior of LLN+ as Stage III disease in the West. In contrast, the current study was carried out in comparable patient groups who were all treated in the West, all receiving nCRT.

There are three studies suggesting that the current Western treatment of nCRT followed by TME is effective to achieve local control in LLN+ patients. To date, one study has been performed that is comparable to the current study (17). In their study, Dharmarajan et al., included a total of 53 patients and showed no difference in 5-year LRFS, DFS, and OS between LLN− and LLN+ patients undergoing nCRT followed by TME surgery. Based on these results, they concluded that in LLN+ patients a LLND is not justified as it is unlikely to provide a survival benefit. However, their study was a single-center experience reporting on a small number of patients only, meaning it was likely underpowered to detect differences in long-term oncological outcomes between LLN+ and LLN− patients. Also, due to the small number of patients, they were unable to perform a propensity score matching analysis to correct for differences baseline criteria. Interestingly though, the DFS and OS rates they reported in both groups were worse than in our study, possibly the result of the more advanced cT and cN-stages. In addition, Syk et al. performed a retrospective cohort study, suggesting that LLN+ are not a major cause of LR after TME (28). Finally, the MERCURY study included 325 patients, in which LLN+ patients were compared to LLN− patients, and showed that LLN+ on pre-treatment MRI have little impact on outcome if nCRT is administered (29). However, both studies also included patients with lower stages of disease (AJCC Stage I and II) and only a portion of the patients underwent nCRT. Furthermore, neither study performed a propensity score matching or multivariate analysis.

On the contrary, other studies, mostly originating from the East, have suggested that nCRT followed by TME alone may not be sufficient treatment for LLN+ (11, 12). For instance, three studies showed that in 33–66% of the LLN+, metastases were found during pathological examination when a LLND was performed after nCRT (12–14). Interestingly, the study by Ishihara et al. reported a 0% LR rate in LLN+ patients, which is considerably lower compared to the current and previous studies reporting on LR rates in LLN+ patients treated with nCRT + TME only (11, 13, 26). Furthermore, they also reported an improved 5-year OS rate of 81.2%, compared with 69% in the current study. However, the median follow-up in their study was considerably shorter: 39 months compared to 58 months in the present study, creating potential bias. Nonetheless, their study, combined with the low LR rates from other recent studies suggest favorable results when a LLND is combined with TME after nCRT, but it remains unclear if these results can be extrapolated to Western patients (12, 26).

Some limitations of the current study have to be addressed. Firstly, this is a retrospective cohort conducted at multiple centers, resulting in heterogeneity of patients and treatment modalities, such as the n(C)RT and adjuvant chemotherapy regimens used. Also, it was clear that LLN+ were not treated the same as mesorectal nodes, with surprisingly few patients receiving adjuvant chemotherapy in the LLN+ group. This was largely due to difference in practice between countries, where in the Netherlands, chemotherapy is mostly reserved as palliative or induction treatment prior to further surgery if a patient develops recurrent disease. In an attempt to overcome these issues, we conducted propensity score matching leading to comparable groups at baseline. However, we did not correct for the MRI-height of the tumor, since the occurrence of LLN+ is closely related to the anatomical height of rectal cancer (22, 30). As a result, the type of nCRT and type of surgery also remained significantly different as these variables are also related to tumor height. We also excluded from the matched cohort patients in whom lateral lymph nodes were selectively removed and all R1 and R2 resections, since independently of the lateral lymph node status, non-radical resections are associated with worse LRFS, DFS and OS and thus would severely influence the long-term oncological outcomes (31, 32). Based on previous publications, a cut-off short-axis size of ≥5 mm for LLN+ was chosen, however, in literature the definition of a LLN+ varies between 5–10 mm for the short-axis, making comparisons challenging (10, 14, 18, 22, 26, 30, 33). Also, we were not able to evaluate the response of the LLN to nCRT as most patients did not undergo a restaging MRI. Finally, although we included patients from five large international tertiary referral centers, the study population was relatively small and potentially underpowered for survival outcomes. In the future, larger multi-center collaborations are required to answer the questions posed in this study using a larger prospective dataset.

In the future, a study comparing Western LLN+ patients undergoing a LLND after nCRT to patients undergoing nCRT only would be important and such a study is currently being undertaken by our group. A recent study by Malakorn et al. showed that a LLND may only be advantageous in patients with persistent LLN+ (>5 mm) on restaging MRI after nCRT. The soon to open Lateral Nodal Recurrence in Rectal Cancer (LaNoReC) study will also be of great interest (34).

In conclusion, in Western patients with Stage III low rectal cancer, there is a trend toward worse LR and DFS rates in LLN+ patients compared to similarly staged LLN− patients. These results suggest that LLN+ patients may currently not be treated optimally with nCRT alone, and the addition of LLND requires further consideration.

## Data Availability Statement

The datasets generated for this study will not be made publicly available. Data cannot be shared as per Ethics approval.

## Ethics Statement

The study was approved by the human research ethics committee at each site. Written informed consent for participation was not required for this study in accordance with the national legislations and the institutional requirements.

## Author Contributions

AH, HK, MK, and TS contributed conception and design of the study. AH, HK, DS, HL, JL, MV, HR, GB, and MK organized the database. AH, HK, and SB performed the statistical analysis. AH and HK wrote the first draft of the manuscript. DS, SB, ND-V, HL, MT, JL, MV, HR, GB, MK, and TS wrote sections of the manuscript. All authors contributed to manuscript revision, read, and approved the submitted version.

### Conflict of Interest

The authors declare that the research was conducted in the absence of any commercial or financial relationships that could be construed as a potential conflict of interest.
